# Under pressure: a critical view on stress-test protocols and cutoffs for anomalous aortic origin of a coronary artery evaluation

**DOI:** 10.1093/ehjimp/qyag059

**Published:** 2026-04-06

**Authors:** Marius Reto Bigler, Anselm W Stark, Lorenz Räber, Christoph Gräni

**Affiliations:** Department of Cardiology, Inselspital, Bern University Hospital, University of Bern, Bern,Switzerland; Department of Cardiology, Inselspital, Bern University Hospital, University of Bern, Bern,Switzerland; Department of Cardiology, Inselspital, Bern University Hospital, University of Bern, Bern,Switzerland; Department of Cardiology, Inselspital, Bern University Hospital, University of Bern, Bern,Switzerland

**This Letter refers to ‘Adults with anomalous aortic origin of a coronary artery: impact of invasive functional testing on clinical decision making-insights from the MuSCAT registry’, by D.B.H. Verheijen *et al*., https://doi.org/10.1093/ehjimp/qyag010.**

We read with great interest the manuscript by Verheijen *et al*.^1^ reporting insights from the prospective MuSCAT (Multicentre Study on Coronary Anomalies in The Netherlands) registry on adults with anomalous aortic origin of a coronary artery (AAOCA). The authors are to be congratulated for assembling a national, multicentre cohort and for systematically integrating non-invasive stress testing with invasive functional assessment using coronary physiology and intravascular ultrasound, both at rest and during pharmacological stress. The key observation—a 28% discrepancy between non-invasive and invasive functional testing with consequent changes in management recommendations in 20% of patients—highlights both the clinical relevance and the current uncertainty surrounding functional significance in AAOCA.

Several methodological aspects deserve attention when interpreting invasive functional results and discordance with non-invasive testing in this specific pathophysiologic setting. First, the invasive stress testing in MuSCAT was not standardized, which limits interpretability of discordance. Although this real-world heterogeneity reflects contemporary clinical practice, it also provided an opportunity to discuss the relative merits of invasive stress protocols using either a dobutamine–atropine–volume challenge^2^ or adrenaline infusion. The inotropic-effect of dobutamine is mediated primarily through β1-adrenoreceptors, enhancing myocardial contractility with a comparatively moderate increase in heart rate resulting in an augmented stroke volume, cardiac output and myocardial oxygen consumption, thereby reproducing the haemodynamic conditions observed during physical exertion. To compensate for the shortcomings of dobutamine alone, it is recommended to add atropine which exerts a pronounced positive chronotropic effect, while volume administration counteracts the β2-mediated peripheral vasodilation induced by dobutamine. Adrenaline, on the other hand, has comparable activity on α1- and β-adrenergic receptors increasing systemic vascular resistance, cardiac output and consequently arterial blood pressure, while also increasing myocardial oxygen consumption. In AAOCA with its unique pathophysiology, myocardial ischaemia arises not only from a fixed anatomical narrowing known from coronary artery disease (CAD), but also from a dynamic component that becomes haemodynamically significant primarily under conditions of strenuous physical exertion. Thus, the optimal diagnostic stress protocol should mimic this prerequisite either by directly applying physical stress or by pharmacological simulation. While there exists no data comparing the different pharmacological stress tests in AAOCA and both protocols have their advantages, the application of a standardized stress test may improve the comparability of the results. Furthermore, and considering the slightly more pronounced increase in myocardial oxygen consumption and coronary blood flow,^3^ application of a dobutamine-atropine-volume challenge rather than adrenalin could provide diagnostics advantages.

Second, the use of standard invasive thresholds for the pressure-derived haemodynamic indices, i.e. fractional flow reserve (FFR) or instantaneous wave-free ratio (iFR) are not validated for AAOCA. While it is common to use the FFR threshold of ≤0.8 as definition for haemodynamic relevance derived from large trials in CAD, there is no specific evidence confirming that the unique pathomechanism in AAOCA can be evaluated using the same criteria. Nevertheless, despite the distinct pathophysiological mechanisms between AAOCA and CAD, the well-established favourable outcomes in CAD patients with FFR values above the ischaemic threshold suggest that the risk of major adverse cardiac events in AAOCA with similarly preserved FFR is reasonably low.^2^ This necessary assumption is currently further extended for non-hyperaemic parameters, i.e. iFR, for the validation of AAOCA under stress conditions as shown in the present study. Initially, iFR was introduced and validated against FFR as a vasodilator-free alternative. It is based on the premise that during the diastolic wave-free period in rest—when microvascular resistance is naturally stable and minimized—the ratio of distal coronary pressure to aortic pressure accurately reflects the haemodynamic relevance of a coronary lesion.^4^ However, in MuSCAT, iFR was not only assessed at rest but also during pharmacological stress to assess the dynamic stenotic component in AAOCA, and classified according to the conventional cut-offs (i.e. ≤0.89 iFR). Mechanistically, non-hyperaemic pressure ratios are sensitive to changes in microvascular resistance; pharmacologic vasodilatation and inotropic/chronotropic stimulation can therefore shift iFR values independently of the extent of the epicardial stenosis severity. Indeed, adenosine-mediated measurements yield systematically lower iFR values than resting iFR, underscoring that iFR is not invariant across physiological states.^4^ Similarly, under dobutamine stimulation, iFR has been shown to decrease substantially in documented dynamic coronary conditions.^5^ In AAOCA, where the clinical question is explicitly exercise/stress-related ischaemia, applying a resting-derived dichotomous cut-off during pharmacological stress risks misclassification.

In summary, Verheijen *et al.* provide important multicentre evidence that invasive functional testing can meaningfully alter management in adult AAOCA. To maximize interpretability and facilitate adoption, future studies should prioritize (i) standardized reference testing and (ii) AAOCA-specific validation of invasive cut-offs.

## Lead author biography

Marius Reto Bigler (born 1991) is a cardiology fellow at the Department of Cardiology, Bern University Hospital, Switzerland. He received his medical degree from the University of Bern, where he developed an early interest in academic medicine through completion of his Master’s and doctoral theses. Motivated by a strong interest in cardiovascular (patho)physiology and academic medicine, he pursued an MD–PhD, gaining comprehensive exposure to clinical research as well as interventional cardiology. His PhD work focused on the functional assessment of coronary artery diseases using structural and haemodynamic parameters as well as intracoronary electrocardiography, reflecting his broader interests in myocardial perfusion and multimodality cardiac diagnostics. Within this setting, he joined the research group of Prof. Christoph Gräni, head of the specialized coronary artery anomaly clinic at the University Hospital of Bern, and focused on advanced diagnostic strategies for coronary artery anomalies, including the comprehensive work-up discussed in this Letter to the Editor.
